# Evaluation of the effectiveness of a multimodal aspiration prevention system in stroke rehabilitation nursing

**DOI:** 10.1371/journal.pone.0342253

**Published:** 2026-02-12

**Authors:** Xibo Sun, Xiulan Wu, Yamei Zhang, Ming Lu, ChongRui Feng, ChuRong Liu, YouMing Gu, FaHua Lu, LiRong Liu, GengBiao Zhang, Zulin Dou, Zhanhao Liu

**Affiliations:** 1 Guangdong Sanjiu Brain Hospital, Guangzhou, Guangdong, China; 2 Department of Nursing Department, Mingxin Rehabilitation Medical Center, Guangzhou, Guangdong, China; 3 Department of Rehabilitation Medicine, The Third Affiliated Hospital, Sun Yat-sen University, Guangzhou, Guangdong, China; Creighton University, UNITED STATES OF AMERICA

## Abstract

**Objective:**

To evaluate the efficacy of the Multimodal Aspiration Prevention System (MAPS) in reducing aspiration incidence, preventing pneumonia, and improving swallowing function in stroke rehabilitation patients.

**Methods:**

A before-after controlled study was conducted involving 855 stroke rehabilitation patients (408 in the MAPS intervention group, 447 in the historical control group). The intervention group received MAPS, including a three-tier risk warning system, standardized intervention procedures, and multidisciplinary collaboration, while the control group received conventional care. Primary outcomes included overt aspiration incidence, with secondary outcomes assessing stroke-associated pneumonia rates, swallowing function improvement, psychological status, and patient satisfaction.

**Results:**

The MAPS group demonstrated a significantly lower incidence of overt aspiration compared to control group (p < 0.05), with complete elimination of aspiration events achieved by the second month of implementation. Secondary outcomes revealed clinically meaningful improvements following MAPS adoption: stroke-associated pneumonia rates decreased substantially (p < 0.05), psychological assessments indicated significant alleviation of anxiety and depression symptoms (p < 0.05), and patient satisfaction scores reached 99.2%. Although swallowing function showed numerical improvement in the MAPS group, the difference did not reach statistical significance when compared to the control group (p > 0.05).

**Conclusion:**

MAPS effectively reduces aspiration and pneumonia risks while enhancing psychological well-being and patient satisfaction in stroke rehabilitation. Its closed-loop management model demonstrates clinical applicability. Further multicenter studies are warranted to validate long-term benefits.

## Introduction

Stroke is a neurological disorder associated with high incidence and disability rates, imposing a substantial global disease burden. In 2019, the global age-standardized incidence of stroke reached 150.5 per 100,000 population, and stroke often leads to long-term functional impairments. In China, stroke has become the leading cause of death, with approximately 2 million new cases annually and a notable trend toward younger onset age [1,2].

As a common post-stroke complication, dysphagia occurs in up to 75% of patients during the acute phase. It not only significantly impairs quality of life but also predisposes individuals to severe complications, with a well-established association with aspiration pneumonia [3]. Aspiration, defined as the pathological entry of food, saliva, or gastric contents into the subglottic airway, is categorized into overt and silent subtypes. Overt aspiration presents with typical symptoms such as coughing and dyspnea, whereas silent aspiration lacks clinical signs and requires instrumental detection (e.g., imaging or endoscopy). Accumulating evidence indicates that aspiration is a key contributor to stroke-associated pneumonia and substantially increases mortality risk [3–5].

Current clinical practices for aspiration prevention are fragmented, including head-of-bed elevation, dietary modification, and swallowing exercises such as thermal-tactile stimulation and effortful swallowing [6]. These approaches, however, face notable limitations. Conventional interventions frequently rely on empirical experience rather than systematic integration, hindering the implementation of standardized protocols. Moreover, traditional screening methods exhibit low sensitivity for detecting silent aspiration, often leading to missed diagnoses. Another additional concern is that routine rehabilitation training lacks objective feedback mechanisms, which compromises patient adherence and constrains long-term therapeutic efficacy.

Collectively, these challenges underscore an urgent need for a scientific, precise, and clinically operable aspiration-prevention framework to mitigate risks and complications in stroke patients [7]. In recent years, the bundle care model has been progressively adopted to consolidate multiple interventions [8,9]. Nevertheless, this approach remains constrained by notable limitations. First, implementation is predominantly driven by nursing teams, with inadequate integration of multidisciplinary expertise from rehabilitation, nutrition, and medicine, resulting in interventions of insufficient depth and breadth. Second, the management mechanism is fragmented and lacks a closed-loop process that encompasses assessment, intervention, monitoring, and management, thereby hindering individualized dynamic adjustments and continuous quality improvement.

Existing studies highlight multidisciplinary collaboration but offer limited guidance for comprehensive care and lack a practical framework to unify rehabilitation, nutrition, medical treatment, and nursing into a single clinical pathway [10–12]. To address these limitations, our research team developed the Multimodal Aspiration Prevention System (MAPS). MAPS is not just an overlay on bundled care, but establishes a scientific, sustainable, and scalable optimized system. MAPS introduces three principal advancements. First, by establishing a closed-loop management framework, MAPS systematically integrates a multidisciplinary collaboration mechanism into the clinical pathway, achieving full-process coordination from assessment and intervention to monitoring and adjustment, thereby overcoming the limitations of traditional nurse-dominated, unidisciplinary approaches. Second, it introduces standardized decision-making and dynamic adjustment mechanisms, ensuring that interventions are both standardized and personalized. Third, by leveraging a digital tracking system, MAPS enables precise, data-driven management and quality control throughout the entire process.

Currently, there is a scarcity of large-scale clinical validations of MAPS in China, particularly regarding to demonstrating its superiority over conventional care. This study, therefore, aims to evaluate the clinical efficacy of MAPS versus standard care in reducing the incidence of aspiration, preventing aspiration pneumonia, and improving swallowing function post-stroke. The findings are expected to provide evidence-based insights for optimizing aspiration management strategies and may facilitate the establishment of standardized clinical protocols to enhance rehabilitation quality.

### Patients and Methods

This study employed a historically controlled, before-after design to evaluate the clinical impact of MAPS. This design was selected for its feasibility in assessing the initial effectiveness of a complex, systems-level intervention in a real-world clinical setting, where randomized controlled trials may be logistically challenging to implement initially.

To mitigate potential biases inherent in this design, several key strategies were implemented. First, we defined two distinct, consecutive 6-month enrollment periods to minimize overlap in clinical care protocols between groups. Second, identical, stringent inclusion and exclusion criteria were applied to both cohorts to ensure population comparability. Additionally, there were no large-scale staff rotations during the study period that could have systematically affected care delivery. Finally, we adjusted for key baseline covariates, including age, stroke type, and sex, in the primary analyses to control for residual confounding.

### Patients

All patients meeting the inclusion criteria were prospectively enrolled in this study. The inclusion criteria were: 1) patients were over eighteen years; 2) diagnosis of stroke according to the diagnostic criteria established in either the Chinese Guidelines for Diagnosis and Treatment of Acute Ischemic Stroke or the Chinese Guidelines for Diagnosis and Treatment of Intracerebral Hemorrhage, with confirmation by cranial CT or MRI [13,14]; 3) in the subacute stage of stroke recovery; 4) conscious and able to cooperate with the study procedures. Exclusion criteria were: 1) concomitant severe dysfunction of the heart, liver, or kidneys; 2) cognitive impairment or psychiatric illness that precluded cooperation; 3) pregnant or lactating women; 4) participation in other interventional clinical studies.

For the historical control group, data were retrospectively collected from electronic medical records of stroke inpatients admitted from December 1, 2023 to May 31, 2024. Trained research staff systematically extracted outcome measures, including the incidence of aspiration, occurrence of aspiration pneumonia, and improvement in swallowing function during hospitalization.The intervention group comprised stroke patients hospitalized between June 1, 2024 and November 30, 2024. After obtaining written informed consent at admission, these patients received the standardized MAPS protocol, and all outcome measures were prospectively recorded by dedicated research personnel.

The study adhered strictly to the ethical principles outlined in the Declaration of Helsinki. The research protocol was reviewed and approved by the Ethics Committee of Mingxin Rehabilitation Medical Center (Approval No. 2024−01). All patient identifiers were anonymized to ensure data security and privacy protection. For patients in the historical control group, the ethics committee granted a waiver of informed consent. Written informed consent was obtained from all participants in the intervention group.

### Multimodal aspiration prevention system

A systematic, multidisciplinary aspiration risk management protocol was implemented to provide comprehensive intervention for hospitalized stroke patients. Upon admission, all patient received a standardized assessment conducted by designated nurses to precisely identify individuals with high or extreme risk of aspiration.

**Fig 1:**
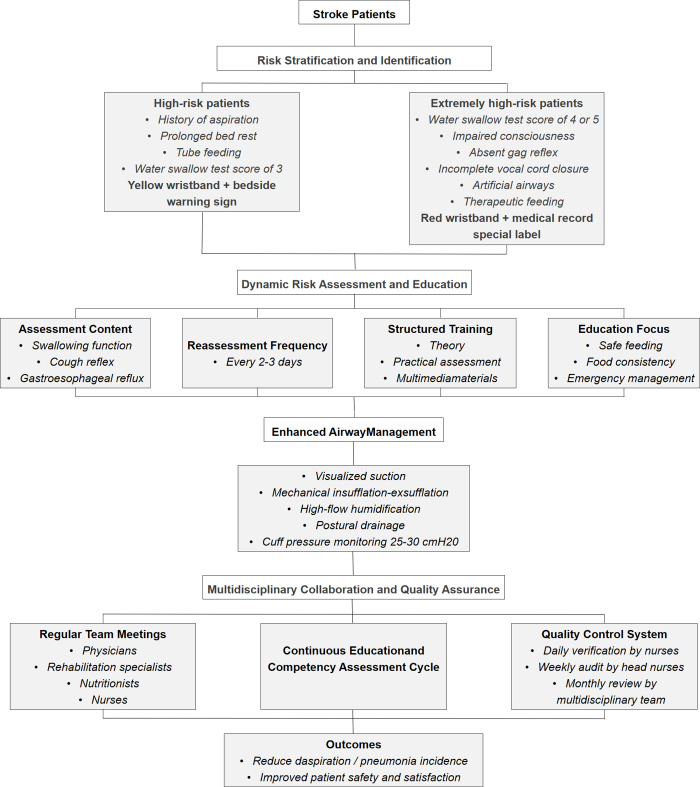
The schematic summary of MAPS.

MAPS protocol integrates four core components: risk stratification using visual identifiers, dynamic assessment of swallowing function and cough reflex, structured patient education covering safe feeding practices and tube feeding management, and enhanced airway care techniques. Implementation fidelity was maintained through a multi-tiered supervision framework and regular interdisciplinary evaluations. Complete operational specifications are available in the S1 File. The schematic summary of MAPS is shown in Fig 1.

### Outcome measures

This study employed a multidimensional approach to evaluate intervention effectiveness. The primary outcome was the incidence of overt aspiration, which was strictly defined as episodes presenting with characteristic clinical manifestations such as coughing, choking, or oxygen desaturation. The silent aspiration was not inclued in the primary outcomes for two reasons. The historical control group relied on past medical records, and there are challenges in clinical detection with a high likelihood of underdiagnosis for silent aspiration, making retrospective ascertainment of silent aspiration unreliable. To maximize consistency between groups, overt aspiration, which is both clinically identifiable and objectively documented, was used as the assessment metric. Although silent aspiration is clinically meaningful, focusing on overt aspiration enhances the reliability of between-group comparisons when comprehensive instrumental screening and historical data are unavailable.

Secondary outcomes encompassed several clinically relevant measures. Stroke-associated pneumonia incidence during hospitalization was documented, diagnosed according to the Chinese Consensus Statement on Stroke-associated Pneumonia [15], explicitly excluding any pre-existing pulmonary infections present at admission. Swallowing function improvement was assessed using the standardized water swallow test administered by certified swallowing therapists prior to discharge, with successful improvement defined as at least a one-grade enhancement compared to baseline evaluation. Patient satisfaction was evaluated using a validated hospital-developed questionnaire that assessed nursing service quality, health education effectiveness, and treatment outcomes on a 100-point scale. Psychological status changes were measured using both the Self-rating Anxiety Scale and Self-rating Depression Scale, with the difference between admission and discharge scores representing the degree of emotional improvement.

To ensure consistency in outcome determination and minimize measurement bias, all primary outcomes were defined using uniform operational criteria. Before study initiation, all personnel involved in data collection and outcome adjudication underwent standardized training, covering case definitions, the use of assessment tools, and procedures for identifying adverse events. The same set of determination standards and procedures was maintained throughout the data collection periods for both the historical control group and MAPS intervention group. All outcome measures were independently collected and cross-verified by two trained researchers to ensure data reliability, with discrepancies resolved through consensus discussion with a third investigator. This approach maintained rigorous data accuracy throughout the study.

### Statistical analysis

Statistical analyses were performed using SPSS version 20.0 (IBM SPSS Inc., Chicago, IL, USA). Continuous variables were assessed for normality using appropriate statistical tests. Data conforming to normal distribution were presented as mean ± standard deviation (M ± SD), while non-normally distributed data were expressed as median with interquartile range [M (IQR)]. Categorical variables were summarized as frequencies and percentages. In data extraction and collation, missing values were found in psychological status. Sensitivity analysis showed that the missing values were completely at random, and median imputation was subsequently applied to impute the values [16].

Comparisons between the MAPS intervention group and historical control group were conducted using statistical tests appropriate to data characteristics. Categorical variables were analyzed using Chi-square tests, normally distributed continuous variables were compared using independent samples t-tests, and non-normally distributed continuous variables were evaluated using Mann-Whitney U tests. Multivariable logistic regression was used to assess the independent effect of MAPS intervention on primary outcomes after adjusting for age, sex, and stroke type. Results are reported as adjusted odds ratios with 95% CIs. All statistical tests were two-tailed, with a p-value < 0.05 considered statistically significant.

## Results

### Demographic characteristics

The study included a total of 855 patients. Baseline characteristics including gender, age, stroke types, and time from onset to admission showed no statistically significant differences between groups (p > 0.05, Table 1).

**Table 1:** Comparison of baseline data between intervention group and control group.

Variable	Experimental N = 408	Control N = 447	Tests statistic	P
Gender (man/ woman)	223/ 185	241/ 206	0.05	0.818
Age (years)	66.3 ± 4.8	65.9 ± 5.2	1.12	0.263
Stroke types (Ischemic/ Hemorrhagic stroke)	219/ 189	237/ 210	0.03	0.850
Onset-to-door time (days)	24.6 ± 3.5	25.1 ± 4.2	1.87	0.062

### Primary outcome

**Fig 2:**
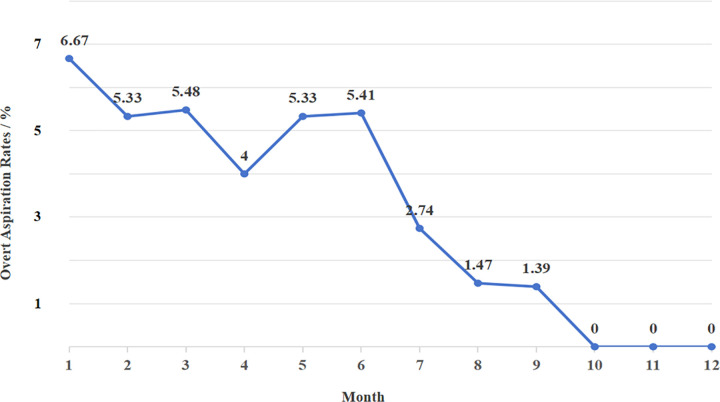
Line chart of monthly changes in overt aspiration rates. The data show the overt aspiration rates for the control group (Months 1–6) and the experimental group (Months 7–12).

The intervention group exhibited a significantly lower incidence of overt aspiration compared with the historical control group (p = 0.002, Table 2). As shown in Fig 2, the aspiration rate in the intervention group declined progressively after MAPS implementation, reaching zero by month four and remaining stable thereafter.

**Table 2:** Comparison of outcomes between intervention group and control group.

Variable	Experimental N = 408	Control N = 447	Tests statistic	Effect Size	P
Cases of overt aspiration	4	24	12.96	0.17(0.05 0.52)	0.002
Cases of stroke-associated pneumonia	42	91	4.07	0.45(0.30 0.67)	<0.001
Cases of improved swallowing function	369	396	0.86	1.22(0.79 1.88)	0.390
SAS	15.2 ± 3.8	13.5 ± 2.9	7.39	0.50 (0.35 0.65)	<0.001
SDS	18.6 ± 4.2	10.1 ± 3.1	33.86	2.30 (2.10 2.50)	<0.001
Satisfaction scores	99.2 ± 2.1	93.2 ± 5.3	4.81	1.55 (1.39 1.71)	<0.001

SAS: Self-rating Anxiety Scale; SDS: Self-rating Depression Scale.

### Secondary outcomes

Following MAPS implementation, several secondary outcomes improved significantly: the incidence of stroke-associated pneumonia decreased markedly, anxiety and depression scores improved significantly, and patient satisfaction scores increased substantially (p < 0.001, Table 2). By contrast, the improvement in swallowing function, although evident numerically, did not reach statistical significance (p > 0.05, Table 2).

## Discussion

This study provides a comprehensive evaluation of MAPS in stroke rehabilitation care. The findings demonstrate significant clinical benefits of MAPS in reducing aspiration events and related complications, offering robust evidence to support aspiration management strategies for stroke patients. The innovative aspect of this research lies in developing a complete closed-loop management system that integrates risk assessment, intervention implementation, and quality monitoring into a sustainable nursing model.

The significant reduction in overt aspiration observed in the MAPS group can be deconstructed into a sequential causal pathway initiated by the system’s components. Firstly, the novel three-tier risk warning system, incorporating bedside warning signs, specialized medical record labels, and color-coded wristbands, enabled efficient visual identification of high-risk patients. This directly addressed the common clinical failure of delayed or missed risk identification. Subsequently, this triggered the execution of standardized intervention protocols. The structured tube feeding procedure, for instance, provided nurses with clear, actionable steps to mitigate the identified risk, ensuring consistency and fidelity in care delivery where empirical practices previously varied. Finally, the multidisciplinary collaboration model facilitated dynamic adjustment and optimization of intervention plans through regular case discussions. Therefore, the success of MAPS lies not merely in its individual components, but in their synergistic integration into a coherent “identify-execute-optimize” workflow, which systematically enhanced operational standardization alongside established visual risk management concepts [17,18].

In the context of infection control, the MAPS group exhibited significantly lower rates of stroke-associated pneumonia. The primary mechanism is the reduction of pathogenic microaspiration. This was achieved through a two-pronged approach: visualized suction techniques enhanced airway secretion clearance, while precise cuff pressure maintenance at 25–30 cmH_2_O helped prevent microaspiration [19]. Simultaneously, standardized oral care protocols additionally reduced bacterial colonization risks. The convergence of these measures constitutes the core causal mechanism for pneumonia prevention. This bundled strategy, reinforced by improved nursing adherence post-training, transformed previously reactive airway management into a proactive, multi-layered defense system, aligning with and validating the principles of bundled care [20,21]. Meanwhile, after systematic training, nurses demonstrated greater adherence to airway management procedures, reducing nursing risks associated with improper practices and thereby improving the quality of care.

Our findings robustly support established models of aspiration prevention, which emphasize risk identification, accurate assessment, and multidisciplinary teamwork as foundational [22]. Current evidence indicates that factors such as advanced age, deteriorating swallowing function, and diminished cough reflex increase the risk of silent aspiration by causing pharyngeal muscle weakness and reduced swallowing movements, which in turn compromise pulmonary defense mechanisms and elevate the risk of silent aspiration and related complications [23]. Screening constitutes the primary step in preventing silent aspiration, and nursing staff should conduct systematic screening of swallowing status at hospital admission. Moreover, nurses should play a pivotal role in coordination and communication within multidisciplinary collaboration to ensure the implementation, coordinated execution, and supervision of silent-aspiration prevention and management plans [21,24]. Developing and rigorously applying standardized silent-aspiration prevention protocols may substantially reduce the incidence of aspiration pneumonia.

No significant difference was observed between groups in swallowing function, but the MAPS group showed clear advantages in psychological status and patient satisfaction. Psychological improvement may be related to MAPS intervention, as well as to overall rehabilitation or the natural course of the disease. The systematic health education component may exert positive effects by improving patients’ self-management capabilities. Standardized nursing procedures ensure care quality, and comprehensive, individualized interventions are facilitated by multidisciplinary collaboration. Patient satisfaction rate of 99.2% indicates that MAPS effectively enhances both objective clinical outcomes and subjective patient experiences, aligning with contemporary principles of patient-centered care [25].

The lack of intergroup differences in swallowing function may be related to both limitations in measurement methods and characteristics of clinical implementation. Although follow-up duration affects long-term observation, the key issue is selecting tools that are sensitive and timely enough to reflect swallowing changes. Water swallow tests are practical but have limited sensitivity for detecting silent aspiration and subtle swallowing changes, possibly failing to fully capture the functional improvements achieved by patients receiving MAPS intervention [26]. Future studies could incorporate objective tools such as videofluoroscopic swallowing studies and establish multiple assessment timepoints post-intervention to longitudinally track functional progression. Besides, all participants received standard swallowing rehabilitation, which may attenuate group differences [27]. Therefore, these non-significant results should be interpreted with caution. Beyond measurement bias, real-world implementation factors (such as individualized care, MAPS system engagement, supervision quality, or procedural fidelity) may influence outcomes, and current tools may still lack sensitivity to silent aspiration and subtle functional changes, potentially obscuring MAPS benefits. More rigorously designed randomized controlled trials with additional control groups can be implemented to differentiate the additive effects of structured care and multidisciplinary collaboration in the aforementioned factors in the future.

The findings of this study carry dual implications for both clinical practice and health policy. By standardizing procedures and implementing visual early-warning mechanisms, MAPS provides promising preliminary evidence for aspiration prevention, with its modular design demonstrating potential for adoption across healthcare facilities. The integration of a tiered risk early-warning framework into routine nursing assessments coupled with an automated alert module to enhance implementation efficiency, may reduce training costs and minimize practice variations through its standardized workflow. From a policy perspective, multidisciplinary aspiration prevention programs could be incorporated into stroke care quality metrics. Linking standardized care to reimbursement mechanisms in the future could further incentivize its widespread implementation.

This study has several limitations that point to specific directions for future research. As a single-center, non-randomized controlled trial, it may be subject to selection bias, center-specific effects, and lack of blinding during outcome assessment. The six-month observation period may be insufficient to evaluated long-term effectiveness. Future studies should extend the follow-up duration. Although the sample size was substantial, the absence of formal sample size calculations limits the interpretation of secondary outcomes, underscoring the need for appropriate estimation methods in future work. The lack of cost-effectiveness analysis and systematic comparison with internationally recognized protocols represents an important gap. Given the resource requirements of MAPS, future research should prioritize economic evaluations and direct comparisons with established international standards to clarify its relative advantages within the global aspiration-prevention framework. Finally, although key baseline covariates with multivariate regression were adjusted, this pragmatic clinical investigation retains inherent limitations due to its design. Detailed clinical characteristics such as stroke severity and comorbidities were not fully captured in the statistical adjustments, which may introduce residual confounding that cannot be completely controlled. To enhance the robustness of findings, multicenter randomized trials within regional healthcare networks are recommended. At the study design stage, more clinical characteristics should be systematically collected and incorporated into the analytical models to more precisely evaluate the independent effects of MAPS intervention. Additionally, implementation strategies should be progressively optimized to establish more adaptable prevention pathways.

Although an a priori sample size calculation was not performed in our study, a post hoc power analysis was conducted based on the total sample size (N = 855) and the observed effect size for the primary outcome (overt aspiration, OR = 0.18). With a two-sided alpha of 0.05, the achieved statistical power was greater than 99%, indicating that the sample size was sufficient to detect a statistically significant difference for the primary outcome.

## Conclusions

This study promising preliminary evidence that MAPS can significantly reduce the incidence of overt aspiration and pneumonia in stroke rehabilitation patients, while also improving psychological status and nursing satisfaction through its standardized risk assessment, tiered interventions, and multidisciplinary collaborative model. However, it should be acknowledged that the single-center, non-randomized design and the limited follow-up period of this study impose important limitations on the generalizability of its findings. Consequently, multicenter, randomized controlled trials with long-term follow-up have become a necessary and urgent research priority. Only through rigorously designed, large-scale, multicenter randomized controlled trials can the applicability, long-term efficacy, and health-economic benefits of this protocol be evaluated systematically, thereby providing high-level evidence for diverse medical conditions.

## Supporting information

S1 File Complete Operational Specifications of Multimodal Aspiration Prevention System. (DOCX)
