# Supplementary material for: Evaluation of the effectiveness of a multimodal aspiration prevention system in stroke rehabilitation nursing
Source: PLoS One. 2026 Feb 12;21(2):e0342253. doi: 10.1371/journal.pone.0342253 (PMC12900288; doi:10.1371/journal.pone.0342253)
Supplement: S1 File — (DOCX) [file pone.0342253.s001.docx]

### Multimodal Aspiration Prevention System

**Risk Stratification and Identification**

High-risk patients included those with a history of aspiration, prolonged bed rest, tube feeding, or a water swallow test score of 3. Extremely high-risk patients were defined as those with a water swallow test score of 4 or 5, impaired consciousness, absent gag reflex, incomplete vocal cord closure, artificial airways, or therapeutic feeding. A tiered identification system was implemented using warning signs at the foot of the bed, special labels on medical records, and color-coded wristbands to ensure rapid recognition by the healthcare team. For high-risk patients, risk reassessment was conducted every three days based on clinical condition, while for extremely high-risk patients, risk reassessment ws performed every day or every two days.

**Dynamic Risk Assessment and Education**

The study employed a structured aspiration risk assessment and education protocol featuring regular monitoring of swallowing function, cough reflex, and gastroesophageal reflux using standardized evaluation tools. Comprehensive health education was provided to both patients and caregivers, emphasizing safe feeding techniques, appropriate food consistency selection, and emergency management of choking episodes.

For patients requiring tube feeding, a standardized operational protocol was established encompassing assessment, positioning adjustment, feeding rate control, and close monitoring with documentation. Training was reinforced through theoretical instruction, hands-on skill evaluation, and multimedia educational materials (Figure 1). All nurses required to complete standardized theoretical training and pass practical skills assessment. The education program addressed multiple aspects including optimal feeding environment and posture, swallowing techniques, food texture modification, proper utensil use, feeding procedures, potential complications and their management, and rehabilitation strategies.


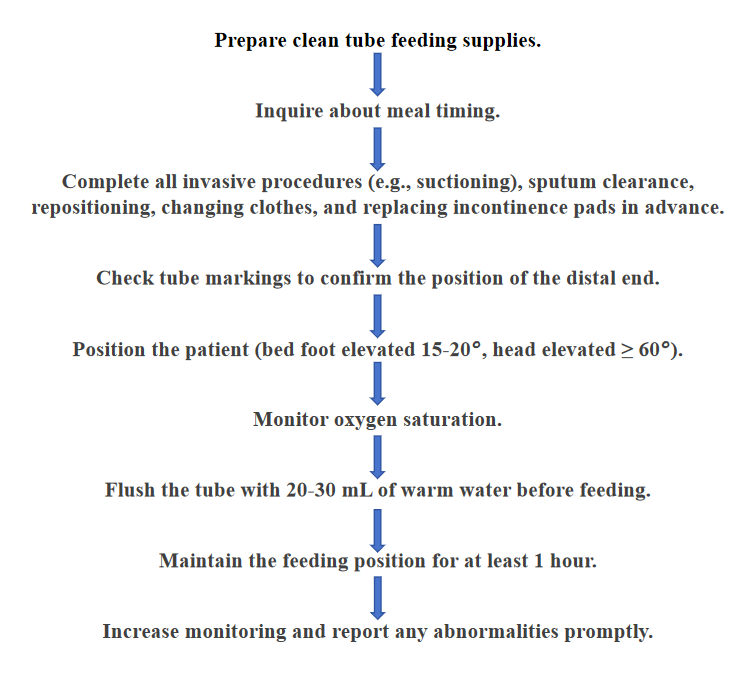


Figure 1. Tube Feeding Procedure

**Enhanced Airway Management**

Tracheostomized patients received comprehensive airway care through a bundled intervention approach. This included visualized suctioning procedures combined with mechanical insufflation-exsufflation techniques for effective secretion management. The protocol incorporated high-flow humidification systems to maintain appropriate airway moisture levels, along with postural drainage and mechanical vibration for secretion mobilization. Meticulous oral care was performed using visualization techniques, while standardized tracheal and subglottic suctioning procedures were implemented with continuous monitoring of cuff pressure, carefully maintained within the 25-30 cmH_2_O range. These integrated measures ensured optimal airway safety through systematic implementation and real-time monitoring (Figure 2).


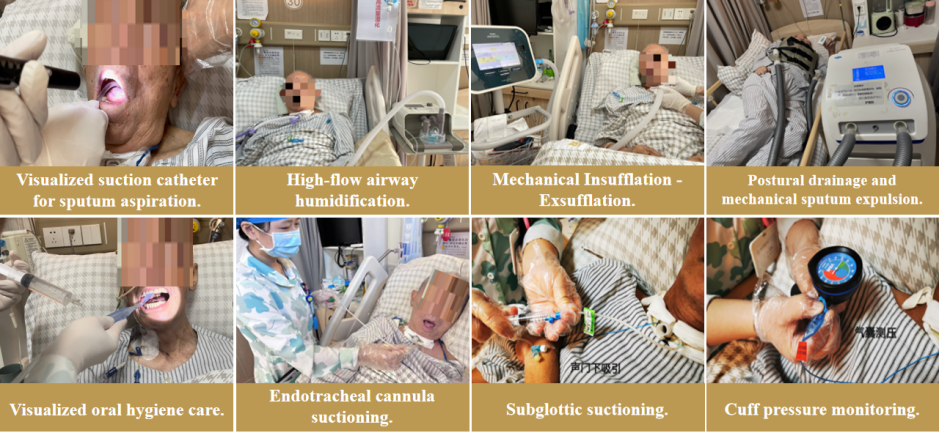


Figure 2. Schematic Diagram of the Airway Care Bundle Management Protocol

**Multidisciplinary Collaboration and Quality Assurance**

Regular interdisciplinary team meetings involving physicians, rehabilitation specialists, nutritionists, and nursing staff were conducted to review patient progress and adjust care plans accordingly. To ensure intervention quality, a quality control system was established: (1) responsible nurses verified the implementation of intervention measures on a daily basis; (2) department head nurses reviewed high-risk patient records and complication data on a weekly basis; and (3) a multidisciplinary quality management team convened monthly to assess outcome indicators, analyze adverse events, and optimize the protocol workflow. Continuous staff education and competency assessment formed an integral part of the quality improvement cycle.
